# SARS Coronavirus Fusion Peptide-Derived Sequence Suppresses Collagen-Induced Arthritis in DBA/1J Mice

**DOI:** 10.1038/srep28672

**Published:** 2016-06-28

**Authors:** Zu T. Shen, Alexander B. Sigalov

**Affiliations:** 1 SignaBlok, Inc, P.O. Box 4064, Shrewsbury, MA 01545, United States of America

## Abstract

During the co-evolution of viruses and their hosts, the viruses have evolved numerous strategies to counter and evade host antiviral immune responses in order to establish a successful infection, replicate and persist in the host. Recently, based on our model of immune signaling, the Signaling Chain HOmoOLigomerization (SCHOOL) model, we suggested specific molecular mechanisms used by different viruses such as severe acute respiratory syndrome coronavirus (SARS-CoV) to modulate the host immune response mediated by members of the family of multichain immune recognition receptors (MIRRs). This family includes T cell receptor (TCR) that is critically involved in immune diseases such as autoimmune arthritis. In the present study, we provide compelling experimental *in vivo* evidence in support of our hypothesis. Using the SCHOOL approach and the SARS-CoV fusion peptide sequence, we rationally designed a novel immunomodulatory peptide that targets TCR. We showed that this peptide ameliorates collagen-induced arthritis in DBA/1J mice and protects against bone and cartilage damage. Incorporation of the peptide into self-assembling lipopeptide nanoparticles that mimic native human high density lipoproteins significantly increases peptide dosage efficacy. Together, our data further confirm that viral immune evasion strategies that target MIRRs can be transferred to therapeutic strategies that require similar functionalities.

The severe acute respiratory syndrome (SARS) coronavirus (SARS-CoV) is the etiological agent of SARS that represents the life-threatening disease associated with a mortality of about 10%^1^. Lymphopenia is observed in most SARS patients with T-helper (CD4^+^) and T-cytotoxic/suppressor (CD8^+^) cell levels reduced in 100% and 87% of the patients, respectively^2^. Most of these patients have reduced CD4^+^ and CD8^+^ cell counts during the early phase of the disease with the lowest cell count values on day 5 and 7 from disease onset^3^,^4^. Like other enveloped viruses encoding class I viral fusion proteins such as human immunodeficiency virus (HIV)^5^ and Ebola and avian sarcoma viruses^6^, SARS-CoV is presumed to use membrane fusion mechanisms for viral entry^7^,^8^. It has been shown that the SARS-CoV viral spike protein 2 (S2) is a class I viral fusion protein that is responsible for driving viral and target T cell membrane fusion^9^. The putative SARS-CoV fusion peptide (FP) has been identified at the N terminus of the SARS-CoV S2 subunit^10^. The fusogenic activity of this peptide has been shown to depend on its amino acid sequence^10^.

Multichain immune recognition receptors (MIRRs) play an important role in the host immune response (reviewed in^11^,^12^,^13^). In MIRRs, the extracellular ligand recognition domains and intracellular signaling sequences containing immunoreceptor tyrosine-based activation motifs (ITAMs) are located on separate protein chains (subunits) bound together by noncovalent transmembrane (TM) interactions^11^,^12^. Structurally, T cell receptor (TCR) is a member of the MIRR family and has the α and β antigen-binding subunits that are bound by electrostatic TM interactions with three signaling homo- and heterodimers: ζζ, CD3εδ, and CD3εγ (Fig. 1a). Short synthetic TM peptides capable of inhibiting TCR-mediated cell activation are known since 1997^14^ when TCR-targeted immunomodulatory activity was first reported for the TCR core peptide (CP), a synthetic peptide corresponding to the sequence of the TCRα TM domain (TMD) known to interact with the TMDs of CD3εδ and ζ^15^,^16^. Similar activity was later reported for HIV FP found in the N terminus of the HIV envelope glycoprotein 41 (gp41)^17^,^18^. Intriguingly, the patterns of TCR-targeted inhibitory activity of TCR CP and HIV gp41 FP were very similar: both peptides inhibit antigen- but not anti-CD3-stimulated T cell activation^18^,^19^. Both peptides were shown to reduce inflammation and ameliorate T cell-mediated autoimmune diseases such as arthritis in animal models^18^,^20^,^21^.

However, despite extensive studies^14^,^17^,^18^,^20^,^21^,^22^,^23^,^24^,^25^, the mode of action of these clinically relevant peptides was enigmatic until a novel model of immune signaling, the Signaling Chain HOmoOLigomerization (SCHOOL) model, was first introduced and applied to this field^13^,^26^,^27^. Previously, using the SCHOOL model and comparative primary sequence analysis of proven and predicted immunomodulatory sequences of viral fusion protein regions, we not only suggested the specific molecular mechanisms of T cell activation inhibition by TCR CP and HIV gp41 FP^27^,^28^,^29^ but also predicted similar immunomodulatory activity for other viral FPs such as SARS-CoV FP (Fig. 1b)^29^.

In this study, we demonstrate that a synthetic 11 amino acid-long peptide (MG11) derived from SARS CoV FP reduces inflammation in DBA/1J mice with collagen-induced arthritis (CIA) and protects mice against bone and cartilage damage. The effect is specific as administration of the control peptide has no effect. Incorporation of MG11 into synthetic nanoparticles that mimic human high density lipoproteins (HDL) substantially reduces the effective peptide dosage. In summary, our data demonstrate for the first time that SARS-CoV FP does not only have fusogenic, but also immunomodulatory activity. This study provides compelling experimental *in vivo* evidence in support of our hypothesis^29^ and further confirms that viral immune evasion strategies evolved during host-virus co-evolution can be transferred to therapeutic strategies that require similar functionalities (e.g., in the treatment of autoimmune diseases).

## Results

### The SARS-CoV FP sequence MG11 reduces inflammation and suppresses the clinical severity of CIA

To evaluate a putative anti-arthritic activity of SARS-CoV FP, we used the SARS-CoV FP-derived peptide sequence MWKTPTLKYFG (MG11). This peptide includes the charge distribution pattern with two essential positively charged amino acid residues (underlined) spaced apart by four amino acids that is similar to that of the TCRα chain TMD either of human (VIGFRILLLKVAGFNLLMTL) or mouse (SVMGLRILLLKVAGFNLLMTL) origin. Based on the SCHOOL model, this sequence has been previously hypothesized to have a similar immunomodulatory activity as TCR CP (GLRILLLKV) or HIV gp41 FP^29^. A SARS CoV FP peptide mutant with two functionally important lysines replaced by glycines (MG11-2G) was used as a negative control peptide. We used the CIA mouse model, the most commonly studied autoimmune model of rheumatoid arthritis (RA)^30^, since a vast majority of the studies of immunomodulatory activity of TCR CP and HIV gp41 FP has been done in animal models of autoimmune arthritis^18^,^20^,^22^,^23^,^31^,^32^.

When intraperitoneally (i.p.) administered daily at a dose of 25 mg/kg, MG11 significantly suppressed arthritis severity compared with administration of vehicle or control peptide MG11-2G (25 mg/kg/day). As shown in Fig. 2a, the difference between the MG11 and vehicle groups started on day 28 and continued until day 38. On day 38, the mean ± SEM clinical arthritis score in MG11-treated mice with CIA was much lower than that in MG11-2G-treated mice (0.89 ± 0.30 versus 3.16 ± 0.47; P < 0.001). The effect is dose-dependent: no anti-arthritic activity was observed for free MG11 i.p. administered daily at a dose of 2.5 mg/kg (data not shown).

Previously, we reported that incorporation of another immunomodulatory peptide, GF9, that employs the SCHOOL mechanisms of action and targets triggering receptor expressed on myeloid cells 1 (TREM-1), into synthetic HDL-like nanoparticles of spherical shape (sHDL) significantly reduces the effective therapeutic dosage of GF9 in animal models of sepsis, lung cancer, and RA^33^,^34^. To evaluate whether incorporation of MG11 into sHDL may have a similar effect, sHDL-bound MG11 was i.p. administered daily at a dose of 2.5 mg/kg MG11. Despite a 10-fold decrease in administration dose of MG11, the arthritis inhibitory effect observed for 2.5 mg/kg/day MG11-HDL was comparable to that observed for 25 mg/kg/day peptide in free form (Fig. 2a). Although the underlying molecular mechanisms of this phenomenon are not completely understood and need to be further investigated, one can suggest that this results from the prolonged circulatory half-life of sHDL-bound MG11: while the *in vivo* peptide half-life is short, typically a few minutes^35^, sHDL are characterized by much longer half-lives up to 3–5 days^36^.

Interestingly, in contrast to vehicle- or MG11-2G-treated mice, administration of MG11 at a daily dose of 25 mg/kg and sHDL-bound MG11 at a daily dose of 2.5 mg/kg resulted in an increase in body weight comparable to that observed for non-arthritic naïve mice (Fig. 2b,c).

In summary, these data collectively indicate that the SARS-CoV FP-derived peptide MG11 generates a strong anti-arthritic effect in the CIA mouse model of RA, thereby providing the first experimental *in vivo* evidence of previously predicted immunomodulatory activity of SARS-CoV FP^29^. Incorporation of MG11 into spherical HDL-like synthetic particles substantially reduces the effective dosage of peptide probably because of the prolonged circulatory half-life afforded by this strategy.

### The SARS-CoV FP sequence MG11 protects against cartilage and bone erosion in CIA

To further evaluate the effect of MG11 in suppressing CIA and determine whether MG11 inhibits chronic inflammation of synovial tissue, pannus formation, cartilage destruction, and bone erosion, we next examined the histopathology of the animal joints (Fig. 3). Overall, mice treated with 25 mg/kg/day free MG11 or 2.5 mg/kg/day sHDL-bound MG11 had significantly lower joint histopathological scores than the vehicle- or MG11-2G-treated groups (*P* < 0.0001) (Fig. 3).

In the vehicle-treated arthritic mice, the fore and hind paw joints had moderate inflammation and cartilage damage with moderate pannus and bone resorption, as well as mild periosteal bone formation, in all joints (Fig. 4). The knee joints had marked inflammation and moderate cartilage damage with pannus formation, bone resorption, and periosteal bone formation (not shown) (Fig. 4). The ankle joints had moderate inflammation and cartilage damage with minimal pannus and bone resorption, as well as mild periosteal bone formation (Fig. 4). Markedly thickened synovial membrane and capsule were observed as a result of pannus formation and inflammatory cell infiltration. As shown in Fig. 4, the chronic inflammation destroyed the joint lining, including the cartilage and other nearby supporting structures, such as bone. The formation of pannus is probably a result of overgrowth of the synoviocytes and the observed accumulation of inflammatory cells that led to deformed cartilage and bone. This agrees with the observed clinical scores. Similar histopathology of the joints was observed in the animals treated with MG11-2G at 25 mg/kg/day (not shown).

For mice treated with MG-11 at 25 mg/kg/day, the fore and hind paw joints had no or very minimal inflammation and minimal cartilage damage (Fig. 4). The knee and ankle joints had no or very minimal inflammation and no or mild evidence of synovial membrane thickening with pannus formation, which falls within normal limits (Fig. 4). Similar histopathology was observed in mice treated with sHDL-bound MG11 at 2.5 mg/kg/day (Fig. 4).

In summary, histopathology examination showed greatly reduced joint inflammation and damage in MG11-treated mice compared with the vehicle-treated mice or mice treated with MG11-2G suggesting a specific protective effect of the MG11 peptide. No significant difference was observed in the histopathological analysis of the joint limbs in mice treated with free MG11 (25 mg/kg/day) or sHDL-bound MG11 (2.5 mg/kg/day). As mentioned above, the prolonged half-life of the peptide incorporated into the HDL particle is probably one of the reasons why sHDL-bound MG11 at a dose of 2.5 mg/kg/day is similarly effective to free MG11 at a dose of 25 mg/kg/day.

### The SARS-CoV FP sequence MG11 reduces cytokine serum levels in mice with CIA

To investigate potential mechanisms underlying the effect of MG11, we examined the serum levels of different cytokines on day 38 using a quantitative Multiplex ELISA array. In mice treated with free MG11 at 25 mg/kg/day or sHDL-bound MG11 at 2.5 mg/kg/day, the cytokine levels were significantly lower than in the vehicle-treated mice or those treated with MG11-2 G at 25 mg/kg/day (Fig. 5). Interestingly, treatment with MG11 reduced the serum level of macrophage colony-stimulating factor (M-CSF) that plays an important proinflammatory role in CIA^37^ and is known to be produced by a variety of cells^38^ including activated T cells^39^,^40^. To further elucidate the molecular mechanisms underlying the observed immunomodulatory effect of MG11 *in vivo*, we used confocal fluorescence microscopy and demonstrated that MG11 inserts into the T cell membrane and colocalizes with TCR in T cells *in vitro* (Supplemental Fig. 1).

In summary, our data suggest that the molecular mechanisms of CIA suppression by MG11 can include inhibition of cytokine and growth factor production mediated by inflammatory T cells that are thought to be central to the pathology of autoimmune arthritis^41^.

## Discussion

To successfully infect, replicate and persist in the host, viruses have evolved numerous strategies to take control of multiple cellular processes including those that target transmembrane signal transduction mediated by immune receptors including MIRRs (reviewed in^42^,^43^,^44^). For T lymphotropic viruses, this approach allows the virus to inhibit TCR signaling to disarm the receptor and successfully enter the cell while for other viruses it allows for evasion from T cell response towards the infected cells^42^,^45^,^46^. Recently reported TCR-targeted immunomodulatory activity mediated by HIV gp41 FP^17^,^18^ suggests that fusion peptides function not only to fuse the virion with the host cell^47^,^48^ but also to silence the TCR signaling pathway. Interestingly, the characteristic pattern of TCR-targeted inhibitory activity of HIV gp41 FP is strikingly similar to that of TCR CP: both peptides colocalize with TCR in the cell membrane, inhibit antigen- but not anti-CD3-stimulated T cell activation *in vitro*, and suppress autoimmune arthritis *in vivo*^18^,^19^,^20^,^21^,^23^,^25^. Both peptides were suggested for the treatment of T cell-mediated pathologies including inflammatory skin diseases and RA^18^,^49^,^50^,^51^. In addition, TCR CP has been shown in human studies to be a proper treatment for human T cell-mediated dermatoses that can substitute for corticosteroids^52^. The molecular mechanisms of action for these clinically relevant peptides were first explained by the SCHOOL model^13^,^27^,^43^,^53^. Later, based on the SCHOOL model and primary sequence analysis of a variety of viral FPs including SARS-CoV FP, we hypothesized that similar to HIV gp41 FP, these FPs may not only have fusogenic but also TCR-targeted immunomodulatory activity and that the SCHOOL model, together with the lessons learned from viral pathogenesis, can be used practically for rational drug design and the development of new therapies for immune disorders^29^.

**Figure 1:**
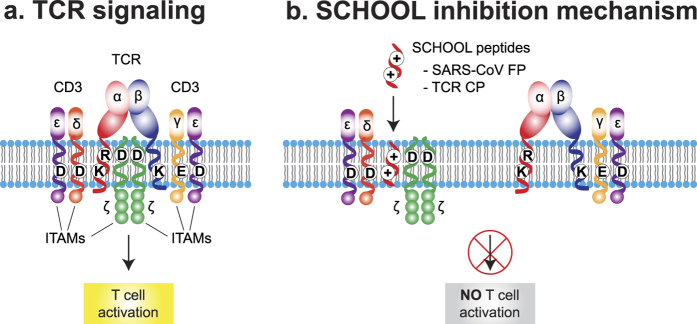
TCR assembly and SCHOOL inhibition mechanism. (**a**) T cell receptor (TCR) assembly is depicted. The TCR α and β recognition subunits are shown in red and blue, respectively. The CD3ε, CD3δ, CD3γ and ζ signaling subunits are shown as purple, dark orange, light orange and green, respectively. Immunoreceptor tyrosine-based activation motifs (ITAMs) are shown as spheres and are colored accordingly by subunit. The recognition and signaling subunits are bound together by electrostatic transmembrane (TM) interactions. These TM interactions occur between basic and acidic amino acid residues. The TCRα transmembrane domain (TMD) contains two basic residues: a lysine, which interacts with two acidic residues of aspartic acid present in the TMDs of the CD3εδ heterodimer, and an arginine, which interacts with two aspartic acid residues present in the TMDs of the ζζ homodimer. The TCRβ TMD contains a lysine, which interacts with one aspartic acid residue and one acidic residue of glutamic acid present in the TMDs of the CD3εγ heterodimer. (**b**) TM-targeted SCHOOL peptides such as the SARS-CoV FP or the TCR CP disrupt TM electrostatic interactions between the TCRα subunit and both CD3εδ and ζζ by competing with TCRα for binding to CD3εδ and ζζ.

As mentioned above, the 19 amino acid-long hydrophobic stretch corresponding to residues 770 to 788 (MYKTPTLKYFGGFNFSQIL) has been recently identified as the putative fusion peptide of the SARS-CoV S2 subunit^10^. In the present study, in order to provide compelling experimental *in vivo* evidence to support our hypothesis^29^, we used the SCHOOL model to design a 11-mer synthetic peptide MG11 (MYKTPTLKYFG) derived from the SARS-CoV FP sequence with the positioning of two essential positively charged lysine residues (underlined) spaced by four amino acids. The model suggests that this charge distribution pattern is functionally important to provide TCR-targeted inhibitory activity^13^,^29^,^42^,^44^. If our hypothesis is correct, the MG11 peptide should demonstrate the immunomodulatory activity *in vivo* similar to that demonstrated earlier for TCR CP and HIV gp41 FP in animal models of autoimmune arthritis^18^,^20^,^21^,^23^. The SARS CoV FP peptide mutant with lysines replaced by glycines (MG11-2G) was used as a negative control peptide. According to the SCHOOL model^13^,^29^, this peptide cannot compete with the recognition TCRα subunit for binding to ζζ and CD3εδ signaling homo- and heterodimers (Fig. 1), and thus cannot inhibit TCR signaling. Because of discrepancies found in prior studies of the immunomodulatory activity of TCR inhibitory TM peptides between *in vitro* (no activity observed) and *in vivo* (anti-arthritic activity observed in rats with adjuvant-induced arthritis, AIA) data^23^, in this study, we moved directly to *in vivo* studies and tested the MG11 and MG11-2G peptides in the CIA model of autoimmune arthritis.

The circulatory half-life of peptides *in vivo* is very short, typically only a few minutes^35^. In order to prolong the half-life of MG11, we tested in the present study whether this peptide can be incorporated into sHDL nanoparticles that mimic human HDL, a group of native lipoproteins that transport cholesterol from the peripheral tissues to the liver and can be readily reconstituted *in vitro* from lipids and apolipoproteins (apos)^54^. Due to the half-life of native sHDL in normal subjects being 3–5 days^36^, these particles represent a promising and versatile delivery platform for peptide therapeutics. Synthetic (reconstituted) HDL have several competitive advantages as compared with other delivery platforms: 1) apo A-I, the major HDL protein, is an endogenous protein and does not trigger immunoreactions, 2) the small size (8–12 nm) allows HDL to enter and accumulate in tissue and organ areas of interest, and 3) a variety of drugs and imaging agents can be incorporated into this platform^33^,^55^,^56^. With respect to therapeutics, human apo A-I is a large protein, which is purified from human plasma. Thus, in addition to the immense monetary cost in purification, further development of apo A-I-containing therapeutic agents would require a number of safety precautions followed by a complicated transition into clinical practice. Previously, we demonstrated that synthetic apo A-I peptides can functionally replace the native apo A-I protein in HDL. This encourages the further development of the HDL-based delivery platform. In the present study, synthetic sHDL that contain apo A-I peptides were successfully loaded with MG11 and subsequently purified and characterized using a variety of biophysical procedures.

**Figure 2:**
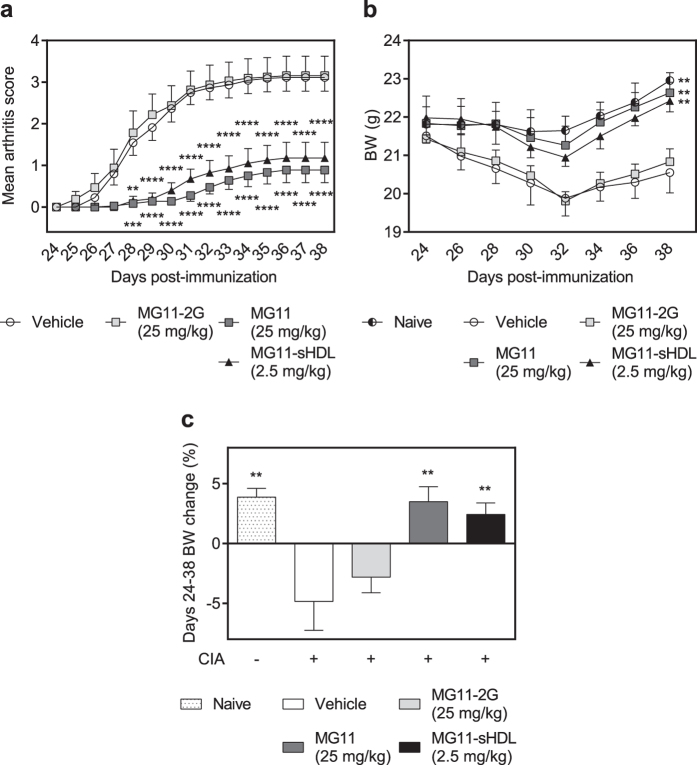
The SARS-CoV FP sequence MG11 strikingly ameliorates the clinical severity of collagen-induced arthritis. (**a**) On day 24 post immunization, different groups of mice with collagen-induced arthritis (CIA) were intraperitoneally (i.p.) administered daily with either vehicle, control peptide MG11-2G (25 mg/kg), MG11 (25 mg/kg) or sHDL-bound MG11 (2.5 mg/kg) for 14 days. Development of arthritis was monitored daily and clinical arthritis was scored. (**b**) Mouse body weight (BW) was measured every other day from day 24 to day 38. (**c**) Percentage in BW change at day 38 compared with day 24. All results are expressed as the mean ± SEM (n = 10 mice per group). **P < 0.01; ***P < 0.005; and ****P < 0.001 versus vehicle.

**Figure 3:**
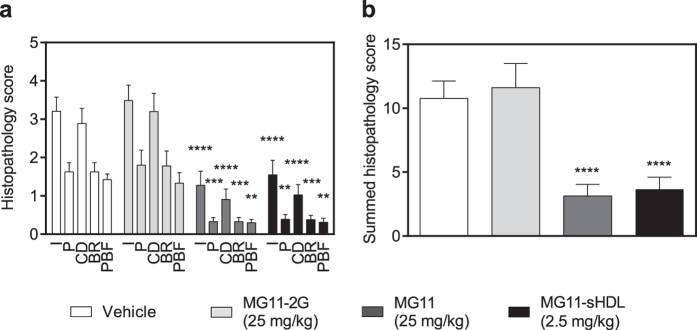
Effects of treatment with the SARS-CoV FP sequence MG11 on joint inflammation, cartilage destruction, pannus formation, and bone resorption in collagen-induced arthritis. (**a,b**) At the end of treatment on day 38, different groups of mice with collagen-induced arthritis (CIA) intraperitoneally (i.p.) administered daily with either vehicle, control peptide MG11-2G (25 mg/kg), MG11 (25 mg/kg) or sHDL-bound MG11 (2.5 mg/kg) were euthanized and evaluated for histopathology. (A) Individual paw and knee joints were scored for inflammation (I), pannus (P), cartilage damage (CD), bone resorption (BR), and periosteal new bone formation (PBF). (**b**) A summed histopathology score, which is the sum of all five histopathological parameters was calculated. All results are expressed as the mean ± SEM (n = 10 mice per group). **P < 0.01; ***P < 0.005; and ****P < 0.001 versus vehicle.

**Figure 4:**
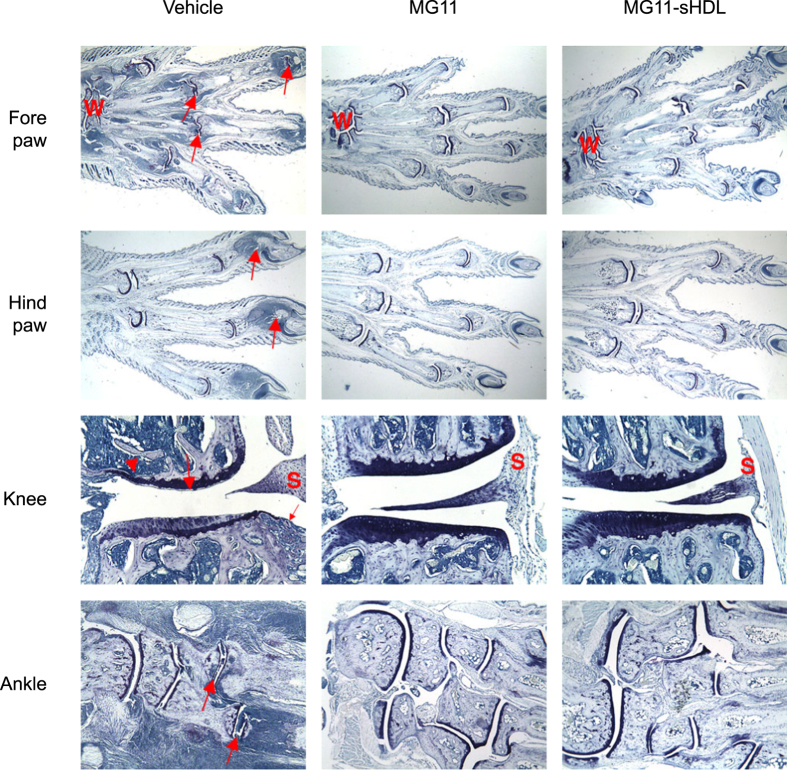
Representative toluidine blue staining of the fore and hind paws and the ankle and knee joints of vehicle- and MG11-treated mice with collagen-induced arthritis. At the end of treatment on day 38, different groups of mice with collagen-induced arthritis (CIA) intraperitoneally (i.p.) administered daily with either vehicle, MG11 (25 mg/kg) or sHDL-bound MG11 (2.5 mg/kg) were euthanized and sections were prepared using fore paws, hind paws, knees and ankles. Individual joint photomicrographs from representative mice are shown for each group. For paws and ankles, arrows identify affected joints. For knees, large arrow identifies cartilage damage, small arrow identifies pannus, and arrowhead identifies bone resorption. W, wrist; S, synovium.

**Figure 5:**
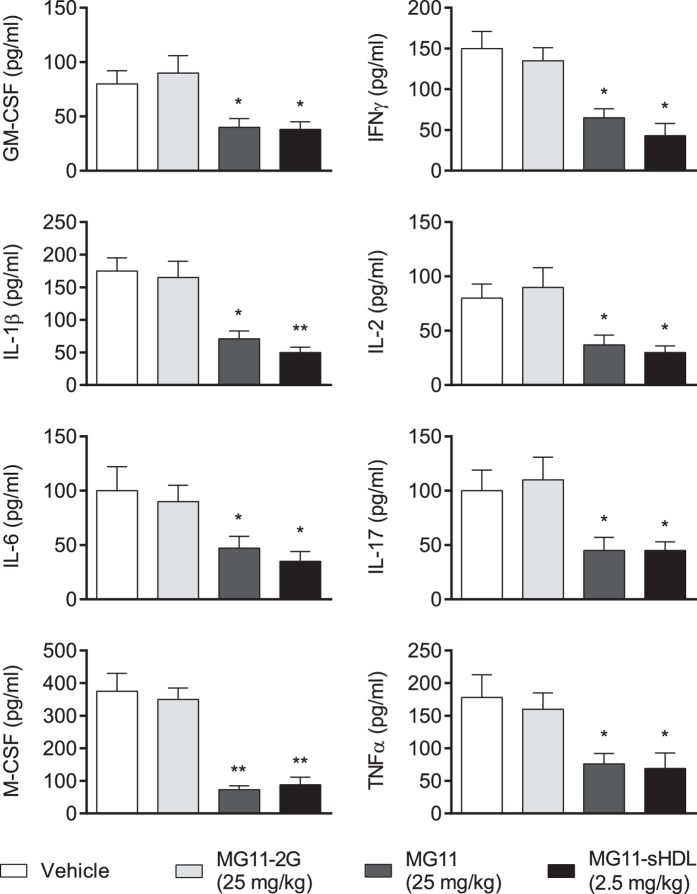
Effects of treatment with the SARS-CoV FP sequence MG11 on cytokine production in collagen-induced arthritis. Serum was collected at the end of treatment on day 38 from different groups of mice with collagen-induced arthritis (CIA) intraperitoneally (i.p.) administered daily with either vehicle, control peptide MG11-2G (25 mg/kg), MG11 (25 mg/kg) or sHDL-bound MG11 (2.5 mg/kg). Serum samples were analyzed for concentrations of granulocyte-macrophage colony-stimulating factor (GM-CSF), interferon-γ (IFNγ), interleukin-1β (IL-1β), IL-2, IL-6, IL-17, macrophage colony-stimulating factor (M-CSF) and tumor necrosis factor-α (TNFα). Results are expressed as the mean ± SEM (n = 5 mice per group). *P < 0.05; **P < 0.01.

This is the first study to test previously predicted immunomodulatory activity of SARS-CoV FP^29^. As expected from the anti-arthritic activities demonstrated in animal models of autoimmune arthritis for TCR CP^20^,^21^,^23^ and HIV gp41 FP^18^, the SARS-CoV FP-derived peptide sequence MG11 significantly suppresses CIA in mice: the peptide at 25 mg/kg/day inhibits inflammation in CIA as assessed by clinical evaluation and scoring of the disease (Fig. 2). Histological analysis of the joints reveals that MG11 substantially reduces joint inflammation, protects against cartilage damage, abrogates bone erosion and reduces systemic bone loss (Figs 3 and 4) The effect is specific as the control MG11-2G peptide administered daily at the same dose of 25 mg/kg does not affect CIA. Incorporation of MG11 into sHDL reduces the effective dosage of the peptide: MG11 in free form at 25 mg/kg/day and sHDL-bound MG11 at 2.5 mg/kg/day show similar anti-arthritic effects in CIA both clinically and histologically. Interestingly, mice treated with free MG11 at a daily dose of 2.5 mg/kg did not exhibit any significant disease improvement as compared to vehicle-treated mice (not shown). At the molecular level, activated T cells mediate production of multiple cytokines and growth factors that are known to be involved in the pathogenesis of RA^57^. Many of these molecules serve as targets of cytokine-blocking therapies that are currently in development (e.g., IL-21, IL-23, and IL-33), at different phases of clinical trials (e.g., IL-7, IL-15, IL-17, and M-CSF) or approved (e.g., TNFα, IL-6, and IL-1 blockers)^57^. In the present study, significantly reduced serum cytokine levels were observed in mice treated with MG11 as compared to vehicle-treated arthritic mice or mice treated with MG11-2G (Fig. 5). Colocalization of MG11 with TCR in the T cell membrane (Supplemental Fig. 1) further supports the suggested molecular mechanisms of the observed immunomodulatory activity of the peptide. These findings are consistent with those previously reported for TCR CP and HIV gp41 FP^18^,^58^.

In summary, the data presented in this study provide the first experimental evidence of the previously predicted immunomodulatory activity of SARS CoV FP^29^ and demonstrate a strong anti-arthritic effect of the SARS CoV FP-derived 11 amino acid-long peptide sequence in a mouse model of RA. Interestingly, immunosuppressive activity of the influenza FP has been recently demonstrated *in vitro*^59^. Further, we suggested before that: 1) short synthetic peptides (SCHOOL peptides) can be designed in line with the SCHOOL platform-based strategy for therapeutic inhibition and modulation of a variety of functionally unrelated multichain receptors expressed on various cells, and 2) the molecular mechanisms of action of the SCHOOL peptides is similar to those that viruses use to evade the immune system^26^,^43^,^44^. To date, the SCHOOL peptides that target TREM-1, glycoprotein receptor VI (GPVI), and TCR were demonstrated both *in vitro* and *in vivo* to represent promising therapeutic approaches to the treatment of a variety of diseases with unmet clinical need including sepsis, lung cancer, rheumatoid arthritis, dermatoses, and others^33^,^34^,^52^,^58^,^60^. Taken together, these findings further support our unifying hypothesis^29^,^44^ that the viral immune evasion strategies developed and optimized during millions of years of evolution of virus-host interactions can be practically used for the rational drug design of new mechanism-based therapies.

## Methods

### Chemicals and lipids

1-palmitoyl-2-oleoyl-sn-glycero-3-phosphocholine (POPC) was purchased from Avanti Polar Lipids (Alabaster, AL). Sodium cholate, cholesterol, cholesteryl oleate and other chemicals were purchased from Sigma Aldrich Company (St. Louis, MO).

### Peptide synthesis

The following synthetic peptides were ordered from American Peptide Company (Sunnyvale, CA): SARS-CoV fusion peptide-derived sequence MWKTPTLKYFG (the SARS-CoV spike glycoprotein S2_770–780_, MG11), control peptide MWGTPTLGYFG (MG11-2G), and two 22-mer peptides PYLDDFQKKWQEEMELYRQKVE (H4) and PLGEEMRDRARAHVDALRTHLA (H6) that correspond to human apo A-I helixes 4 and 6, respectively. Peptides were purified by reversed-phase high-performance liquid chromatography (RP-HPLC), and their purity was confirmed by amino acid analysis and mass spectrometry.

### Spherical lipoproteins

The MG11-containing spherical HDL (MG11-sHDL) complexes were synthesized by the sodium cholate dialysis procedure essentially as described^33^. The molar ratio was 125:6:2:3:1:210 for POPC:cholesterol:cholesteryl oleate:MG11:apo A-I:sodium cholate. Briefly, POPC, cholesterol, and cholesteryl oleate in organic solvents were mixed, dried in a stream of argon, and placed under vacuum for 8 h. Then, lipid films were dispersed in Tris-buffered saline-EDTA (TBS-EDTA, pH 7.4) and sonicated for 5 min. To the dispersed lipids, MG11 in aqueous solution of propylene glycol, ethanol, and Tween-80 was added. The amount of peptide was controllably varied in different preparations. Then, sodium cholate solution was added and the mixture was incubated at 50 °C for 30 min. After cooling to 30 °C, the solution containing a 1:1 mixture of apo A-I peptides H4 and H6 in PBS, pH 7.4 was added and the mixture was incubated at 30 °C for 3 h, followed by extensive dialysis against PBS to remove sodium cholate. The obtained MG11-sHDL particles were then purified on a calibrated Superdex 200HR gel filtration column (GE Healthcare Biosciences, Pittsburgh, PA) using the BioCAD 700E Workstation (Applied Biosystems, Carlsbad, CA) and characterized by analytical RP-HPLC and nondenaturing gel electrophoresis as described previously^33^. Final peptide compositions were determined in the prepared particles by analytical RP-HPLC as previously described^33^. The mean size of the particles was determined using electron microscopy as described^33^.

### Animal studies

Animal studies were performed by Bolder BioPATH (Boulder, CO). All animal experiments were performed in strict accordance with the recommendations in the Guide for the Care and Use of Laboratory Animals of the National Institutes of Health (NIH) and in the United States Department of Agriculture (USDA) Animal Welfare Act (9 CFR, Parts 1, 2, and 3). The protocol (BBP-001.B) was approved by the Institutional Animal Care and Use Committee (IACUC) of Bolder BioPATH for compliance with regulations prior to study initiation (Animal Welfare Assurance number A7649-06) and all methods were performed in accordance with the approved protocol.

Male 6–7 week old DBA/1 mice from Harlan (Indianapolis, IN) were anaesthetized with Isoflurane (VetOne, Boise, ID) and injected intradermally with 100 μL of Freund’s Complete Adjuvant (Sigma Aldrich Company, St. Louis, MO) (2.5 mg/ml final concentration) containing bovine type II collagen (Bolder BioPATH, Boulder, CO) (2 mg/ml final concentration) at the base of the tail on day 0 and again on day 21. On day 24, mice were randomized by body weight into treatment groups. Mice weighed approximately 17–25 grams (mean 20 g) at enrollment on day 24 when treatment was initiated. Mice were i.p. injected with 25 mg/kg/day MG11 or MG11-2G, or 2.5 mg/kg/day MG11-sHDL, or with PBS for 14 days beginning at day 24. Arthritis onset occurred on days 26–38. Mice were weighed on study days 24, 26, 28, 30, 32, 34, 36 and 38 (prior to necropsy). Daily clinical scores were given on a scale of 0–5 for each of the paws (right front, left front, right rear, left rear) on days 24–38 using the following criteria: 0 = normal; 1 = one hind or fore paw joint affected or minimal diffuse erythema and swelling; 2 = two hind or fore paw joints affected or mild diffuse erythema and swelling; 3 = three hind or fore paw joints affected or moderate diffuse erythema and swelling; 4 = four hind or fore paw joints affected or marked diffuse erythema and swelling; 5 = entire paw affected, severe diffuse erythema and severe swelling, unable to flex digits. On day 38, mice were humanely euthanized for necropsy. Mice were anesthetized with Isoflurane and bled by cardiac puncture. Serum was prepared and stored frozen at −80 °C for cytokine analysis.

For histology, fore paws, hind paws, and knees were harvested and placed in 10% neutral buffered formalin (NBF). After 1–2 days in fixative and 4–5 days in 5% formic acid for decalcification, tissues were trimmed, processed for paraffin embedding, sectioned at 8 μm, and stained with toluidine blue (T blue). Hind paws, fore paws, and knees were embedded and sectioned in the frontal plane. Six joints from each animal were processed for histopathologic evaluation. The joints were then assessed for inflammation (0–5 scale), pannus formation (0–5 scale), cartilage damage (0–5 scale), bone resorption (0–5 scale), and periosteal new bone formation (0–5 scale). A summed histopathology score was also determined (sum of five parameters).

### Cytokine analysis

Serum samples were collected on day 38 and cytokines were analyzed using Quantibody Mouse Cytokine Array Q1 kits from RayBiotech (Norcross, GA) following manufacturer’s instructions.

### Statistics

Data analyses were performed using Prism 6.0 (GraphPad Software, Inc., La Jolla, CA). Results are expressed as the mean ± SEM. Statistical differences were analyzed using analysis of variance with Bonferroni adjustment. *P* values less than 0.05 were considered significant.

### Highlights

- Anti-arthritic activity is demonstrated for the fusion peptide of severe acute respiratory syndrome coronavirus (SARS-CoV) *in vivo*.
- The peptide substantially decreases cytokine release *in vivo.*
- Incorporation of the peptide into nanoparticles significantly increases peptide dosage efficacy.

## Additional Information

**Accession codes:** Accession numbers (UniProtKB/Swiss-Prot knowledgebase, http://www.expasy.org/sprot/) for the protein sequences discussed in this Research Article is as the follows: T cell receptor alpha chain, P01848 (human) and P01849 (mouse); SARS-CoV, P59594; HIV-1, P04578.

**How to cite this article**: Shen, Z. T. and Sigalov, A. B. SARS Coronavirus Fusion Peptide-Derived Sequence Suppresses Collagen-Induced Arthritis in DBA/1J Mice. *Sci. Rep.* **6**, 28672; doi: 10.1038/srep28672 (2016).

## Supplementary Material

Supplementary Information
