# Supplementary material for: SARS Coronavirus Fusion Peptide-Derived Sequence Suppresses Collagen-Induced Arthritis in DBA/1J Mice
Source: Sci Rep. 2016 Jun 28;6:28672. doi: 10.1038/srep28672 (PMC4923882; doi:10.1038/srep28672)
Supplement: Supplementary Information [file srep28672-s1.pdf]

# SUPPLEMENTAL FIGURES AND METHODS

## SARS Coronavirus Fusion Peptide-Derived Sequence Suppresses Collagen-Induced Arthritis in DBA/1J Mice

Zu T. Shen, Alexander B. Sigalov*

SignaBlok, Inc, P.O. Box 4064, Shrewsbury, MA 01545, United States of America

* Phone: 203-505-3807. E-mail: sigalov@signablok.com

**Short title:** Anti-Arthritic Effect of SARS-CoV Fusion Peptide

**Highlights:**

- Anti-arthritic activity is demonstrated for the fusion peptide of severe acute respiratory syndrome coronavirus (SARS-CoV) *in vivo*.
- The peptide substantially decreases cytokine release *in vivo.*
- Incorporation of the peptide into nanoparticles significantly increases peptide dosage efficacy.

**Supplemental Figure Legends**

**Supplemental Fig 1. MG11 colocalizes with TCR in the T cell membrane.**

Jurkat T cells were incubated with Alexa 647-conjugated goat anti-mouse antibody following incubation with purified mouse anti-CD3 (OKT3) and Dylight 488-labeled MG11 peptide. After incubation at 4°C, cells were held at 37°C to allow for receptor clustering. Cells were examined by confocal microscopy and a representative image is shown. Colocalization of MG11 (green) with TCR (red) is clearly observed in the merged image (yellow). Bar, 5 μm.

**Supplemental Figure I**



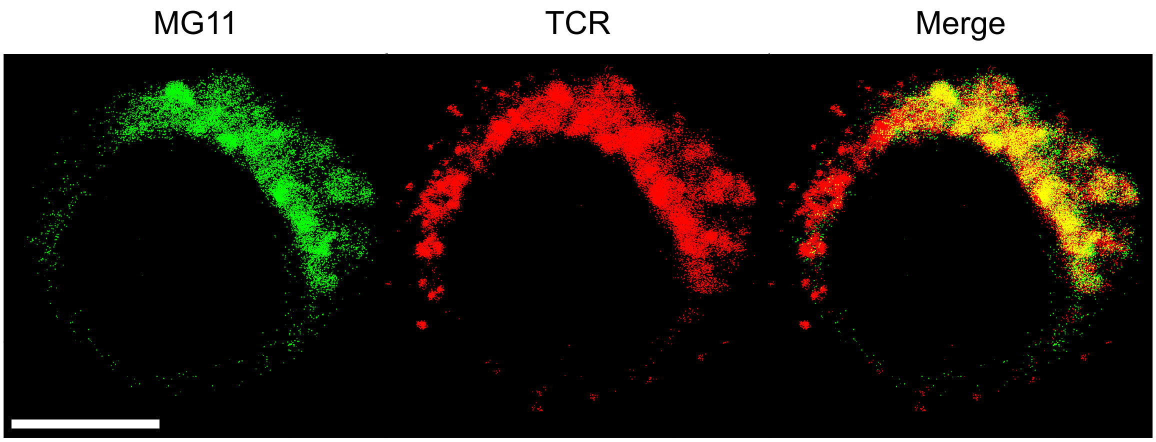

## Supplemental MATERIALS AND METHODS

### Peptides

MG11 peptide was solubilized using 0.1 M phosphate, pH 8 and reacted using a 2-fold molar excess of Dylight 488 N-Hydroxysuccinimide (NHS) ester (ThermoFisher Scientific, Waltham, MA, USA) and incubated at 25°C for 3 h. The reaction was quenched using a 25-fold molar excess of ethanolamine (Sigma-Aldrich, St. Louis, MO, USA) relative to the NHS ester. The reaction mixture was purified using the BioCAD 700E High Pressure Liquid Chromatography (HPLC) system (Applied Biosystems, Carlsbad, CA, USA) fitted with a Vydac 218TP54 C18 reverse phase (RP) HPLC column (Grace Vydac, Hesperia, CA, USA).

### T cells

Jurkat T cells (clone E6-1) were obtained from the American Type Culture Collection (ATCC, Manassas, VA, USA). Cells were cultured at 37°C with 5% $CO_2$ in RPMI 1640 medium supplemented with 10% fetal bovine serum and 100 U $ml^{-1}$ penicillin and streptomycin in 6 well plates (Corning, Tewksbury, MA, USA) containing autoclaved 22 x 22 mm glass coverslips (VWR, Radnor, PA, USA).

To visualize colocalization of MG11 and TCR, Jurkat T cells were first incubated on ice for 10 min. Then, 25 μM MG11 peptide containing a portion of Dylight 488-labeled peptide was added together with 20 μg/ml mouse anti-CD3 OKT3 (Biolegend, San Diego, CA, USA) for 1 h on ice. Next, 10 μg/ml of Alexa 647-labeled goat anti-mouse antibody (Jackson ImmunoResearch Laboratories, West Grove, PA, USA) was added for 1 h on ice. Cells were incubated for 10 min at 37°C to allow receptor capping. Cells were fixed using 4% paraformaldehyde (Alfa Aesar, Heysham, Lancashire, UK) in HBSS for 50 min at 4°C. Then, 50 μl of Prolong Gold antifade mounting medium (Invitrogen, Carlsbad, CA, USA) was used to mount the coverslip to the microscope slide. The

mounted slide was allowed to cure at 25°C in the dark for at least 72 hrs. Mounted slides were stored at 4°C and analyzed by confocal microscopy.

**Confocal microscopy**

Confocal imaging was performed using a Leica TCS SP5 II laser scanning confocal microscope (Leica, Microsystems, Mannheim/Wetzlar, Germany) with acousto-optical beam splitter equipped with hybrid detectors, a detector for transmitted light including differential interference contrast (DIC) and eight laser lines at 405, 458, 476, 488, 496, 514, 561 and 633 nm. Leica Application Suite Advanced Fluorescence software was used to adjust laser and detector settings and to acquire data. Images were acquired using a 63x oil-immersion objective lens, hybrid detectors, a 488 nm laser for Dylight 488, and a 633 nm laser for Alexa 647. Acquired images were analyzed using Leica Application Suite X.
